# Debridement, internal fixation, and reconstruction using titanium mesh for the surgical treatment of thoracic and lumbar spinal tuberculosis via a posterior-only approach: a 4-year follow-up of 28 patients

**DOI:** 10.1186/s13018-015-0292-7

**Published:** 2015-09-22

**Authors:** Hongqi Zhang, Kefeng Zeng, Xinghua Yin, Jia Huang, Mingxing Tang, Chaofeng Guo

**Affiliations:** Department of Spine Surgery, Xiangya Hospital of Central South University, Xiangya Road 87, Changsha, China

**Keywords:** Spine tuberculosis, Posterior-only approach, Specially formed titanium mesh cage, Thoracic and lumbar spine

## Abstract

**Background:**

The standard recommended method for surgical treatment of spinal tuberculosis is an anterior approach for debridement and fusion combined with posterior instrumentation. However, the method has its disadvantages. The aim of this study was to analyze the effectiveness and safety of treating thoracic and lumbar spinal tuberculosis with debridement, internal fixation reconstruction, and using specially formed titanium mesh cages via a posterior-only approach.

**Methods:**

The authors retrospectively reviewed the cases of 28 patients with spinal tuberculosis treated by debridement, internal fixation, and reconstruction with a specially formed titanium mesh cage via a posterior-only approach. The levels involved were less than two contiguous vertebrae: 13 thoracic vertebrae, 5 thoracolumbar vertebrae, and 10 lumbar vertebrae. All patients suffered from back pain, and nine patients had neurologic deficits (two were class C and seven were in class D according to the American Spinal Injury Association classification). All patients were followed up every 3 months after surgery, with a minimum 48-month follow-up. The clinical efficacy was evaluated based on the visual analog scale (VAS), the Oswestry Disability Index (ODI), neurological status, kyphosis angle, and erythrocyte sedimentation rate (ESR).

**Results:**

All patients obtained solid bony fusions without failure of fixation. The infections were resolved in all patients, as noted by normalization of their ESR. The average surgery time was 2 h and 15 min, with an average blood loss of 435 ml. The VAS scores dropped from a preoperative level of 6.31 ± 1.25 to the final follow-up level of 0.57 ± 0.14. The ODI scores dropped from 39.14 ± 12.38 preoperatively to 7.29 ± 3.09 at 1 year postoperatively and 6.77 ± 2.53 at final follow-up. The kyphosis Cobb’s angle was corrected from 22.31° ± 4.26° preoperatively to 5.86° ± 0.57° at final follow-up. No subsidence of titanium mesh cage or posterior instrumentation failure was observed postoperatively. The neurological outcome increased by 1–2 grades in the patients with neurological deficits.

**Conclusions:**

Debridement, internal fixation, and reconstruction using specially formed titanium mesh cages via a posterior-only approach is effective and safe for treating adults with thoracic and lumbar spinal tuberculosis involving less than two contiguous levels.

## Background

Spinal tuberculosis (TB) affects around half of all patients with musculoskeletal TB [1]. The intervertebral disc and the end plates of the adjacent superior and inferior vertebral bodies are often involved in TB; severe destruction of these elements often leads to a kyphotic deformity [2]. This can result in hazardous consequences, especially in the thoracic and lumbar spine with their peculiar anatomy and inherent instability.

The main indication for surgical intervention is the presence of a neurologic deficit, which occurs in around 15–50 % of tuberculous spondylodiscitis patients [3, 4]. Other indications include abscess formation [5], persistent or recurrent infection, severe pain [1], local kyphosis, and segmental instability [2].

Although a single one-stage anterior approach with interbody fusion has been successfully applied in the spine, this method has disadvantages in operation time, blood loss, and hospitalization. In addition, anterior debridement may reduce the biomechanical stability of the spine, and it is common to find residual kyphosis at the end of treatment [6, 7]. Thus, anterior debridement combined with posterior fusion and fixation was developed, which helped to arrest the disease early, provide early fusion, correct kyphosis, as well as prevent its progression [8, 9]. However, the combined procedures are associated with a longer operating time, greater blood loss, more postoperative complications, and longer hospital stay.

The purpose of our clinical study is to investigate the efficacy and safety of treating thoracic and lumbar spinal TB in adults via a posterior-only approach debridement, internal fixation, reconstruction using specially formed titanium mesh cage (TMC) involving no more than two contiguous levels.

## Methods

### Patient population

From March 2009 through March 2010 at our institution, 28 patients with thoracic and lumbar TB with less than two contiguous levels involved were enrolled in the study. There were of 17 men and 11 women. Age at operation (mean ± standard deviation) was 42.73 ± 5.81 years old (range 33 to 68 years). Diagnosis of active TB was made based on clinical symptoms, laboratory findings, and radiographic evidence. Patients who had one of these manifestations as well as *Mycobacterium tuberculosis* infection confirmed postoperatively through laboratory tests such as acid-fast bacillus staining, bacterial cultures, or polymerase chain reaction or pathology were defined as having active spinal TB.

All patients had typical symptoms of TB, including night fever, loss of weight, fatigue, and back pain. Chest radiographs showed the presence of old pulmonary TB in four patients.

Self-reported measures included a ten-point visual analog scale (VAS) to evaluate back pain (Table 1) and the Oswestry Disability Index (ODI).

**Table 1:** VAS, ESR, and radiological examination of patients

	Preoperative	Postoperative	Final follow-up
VAS	6.31 ± 1.25	2.17 ± 1.32	0.57 ± 0.14
ESR (mm/h)	49.27 ± 12.13	20.51 ± 4.13	10.21 ± 3.16
Cobb (°)	22.31 ± 4.26	5.41 ± 0.63	5.86 ± 0.57

**Fig. 1:**
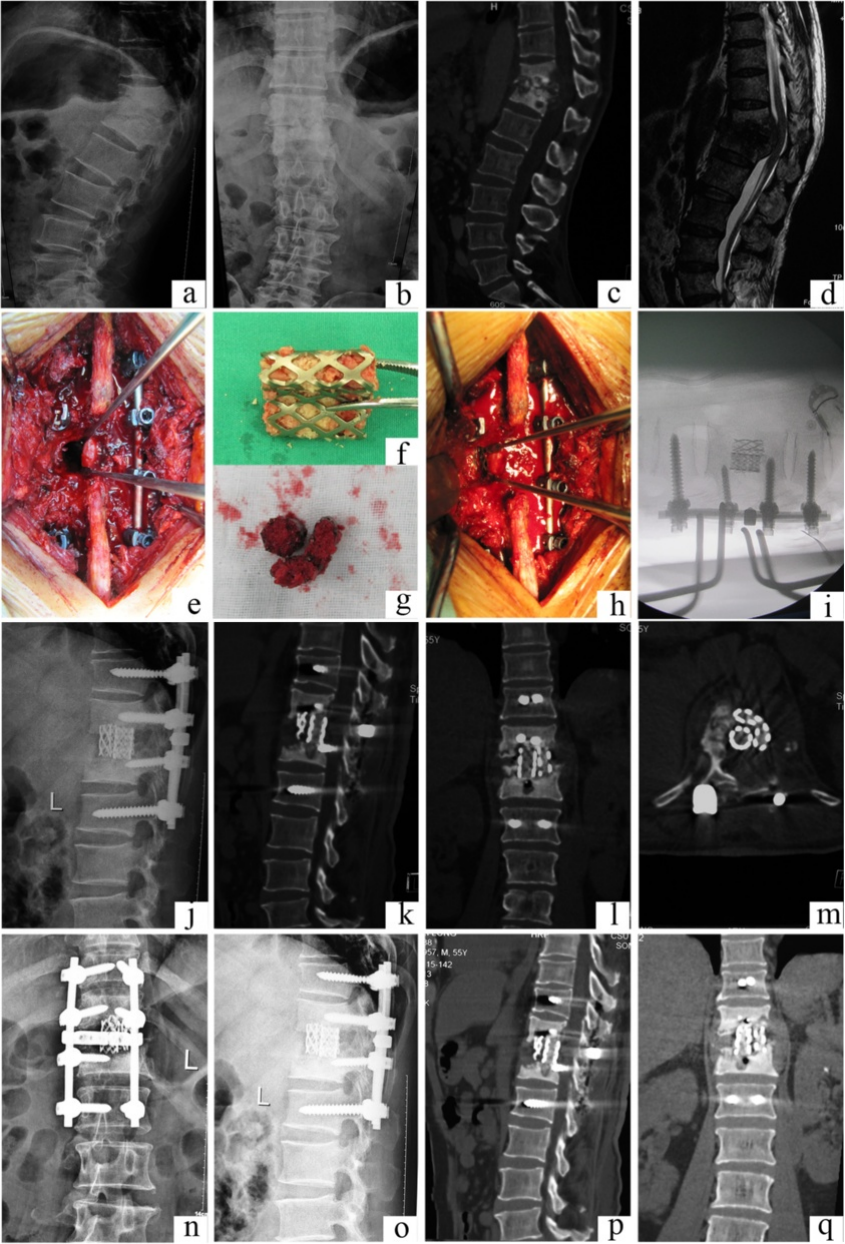
Case number 2. Preoperative image evaluation. **a**, **b** Radiograph showing T11–12 involvement. **c** Computed tomography (CT) and **d** magnetic resonance imagery (MRI). The sagittal T2-weighted MRI showed a T11 vertebral mass with collapse and kyphosis, compressing the spinal cord. **e**–**i** Operation technique and specially formed titanium mesh cages. **j** Postoperative radiograph. **k**–**m** Postoperative CT showing the kyphosis is correct. **n**–**q** The latest follow-up at 48 months postoperatively showed that the internal fixation was in good shape, interbody fusion had been obtained, and there were no signs of tuberculosis recurrence

**Fig. 2:**
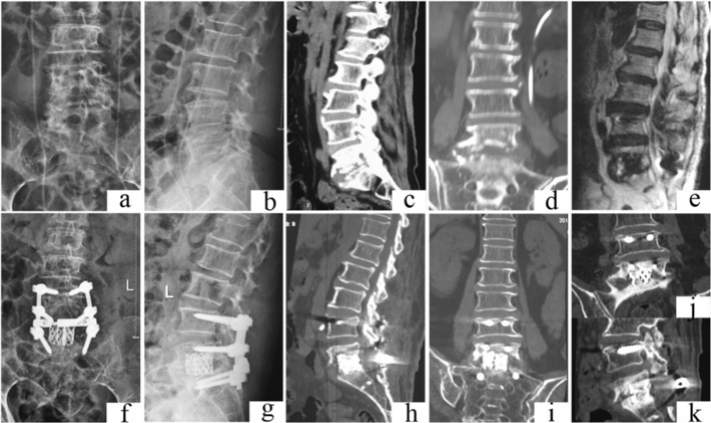
Case number 11. Preoperative image evaluation. **a**, **b** Radiograph showing L5–S1 involvement. **c**–**d** Computed tomography (CT) and **e** magnetic resonance imagery (MRI). **f**–**j** Postoperative radiography and **h**–**i** CT. **j**, **k **At final follow-up, the internal fixation was in good shape and interbody fusion had been obtained, without signs of tuberculosis recurrence

**Fig. 3:**
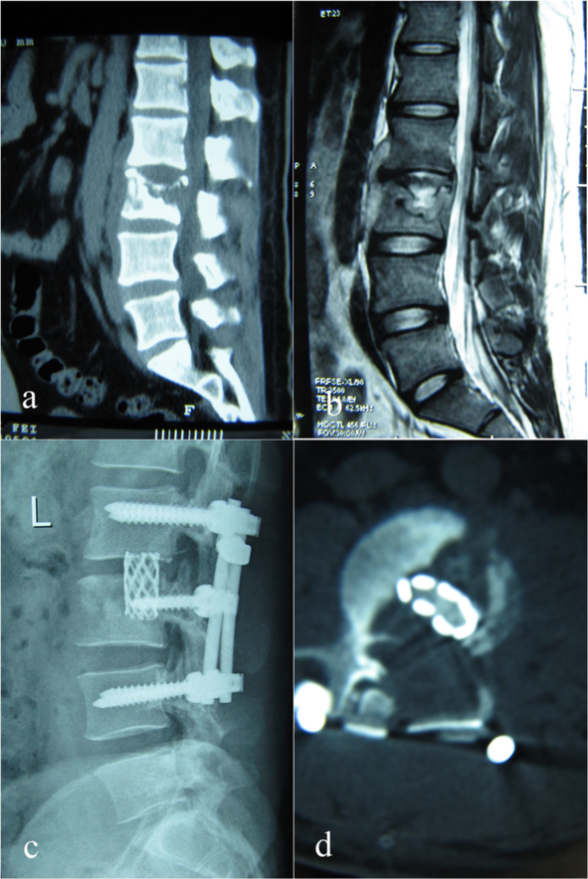
Case number 17. Preoperative image evaluation. **a** Computed tomography (CT) and **b** magnetic resonance imagery (MRI) showing L3 involvement. **c, d** At final follow-up, the internal fixation was in good shape and interbody fusion had been obtained, without signs of tuberculosis recurrence

**Fig. 4:**
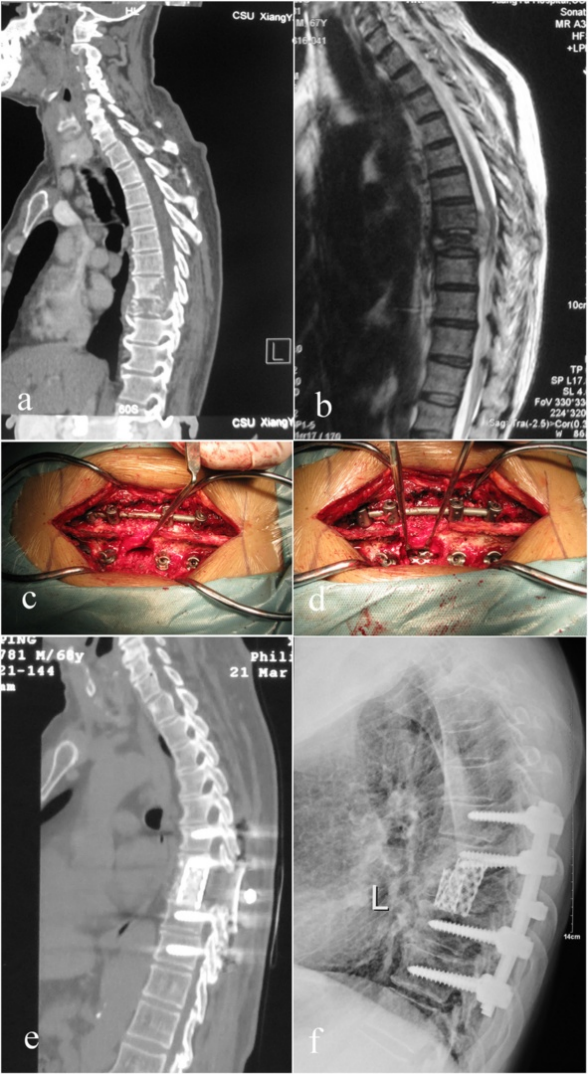
Case number 25. Preoperative image evaluation. **a** Computed tomography (CT) and **b** magnetic resonance imagery (MRI) showing T8–T9 involvement. **c**, **d** Operation technique and specially formed titanium mesh cages. **e**, **f** At final follow-up, the internal fixation was in good shape and interbody fusion had been obtained, without signs of tuberculosis recurrence

The radiographic assessment included preoperative standard anteroposterior and lateral views. All patients had two vertebral bodies involved, and the sagittal profile was measured by Cobb’s method as the angle between the upper end plate and the lower end plate of the infected level (Table 1 and Figs. 1, 2, 3, and 4). The average preoperative Cobb’s angle was 22.31° ± 4.26° (range 13.2°–46.1°).

The neurologic status was graded according to the American Spinal Injury Association (ASIA) impairment scale. All patients had intractable pain due to instability, and nine suffered from evident neurological deficits (Table 2).

**Table 2:** Neurologic recovery according to ASIA

Preoperative		Final follow-up		
		C	D	E
C	2	0	1	1
D	7	0	1	6
E	19	0	0	19

Surgery was indicated if patients had one or more of the following: presence of a neurologic deficit, epidural abscess formation, or local kyphosis with severe end plate destruction and intractable pain.

Written informed consent was obtained from all patients, and the protocol was approved by the Ethics Committee of Xiangya Hospital.

### Preoperative preparation

Patients participating in this study were treated with the HREZ chemotherapy regimen for 2–4 weeks prior to surgery; this consisted of isoniazid (300 mg/day), rifampicin (450 mg/day), ethambutol (750 mg/day), and pyrazinamide (750 mg/day).

### Operative technique

The patients were placed in the prone position following administration of general endotracheal anesthesia. Extraperiosteal dissection was performed through a midline incision to expose the posterior spinal elements, including the laminae, facet joints, and transverse processes; the dissection exposed an adequate number of vertebrae above and below the involved segment(s). Pedicle screws were then inserted under fluoroscopic guidance into the normal vertebrae (two or more above and below the lesion). Bilateral laminectomy was performed at the marked levels so that the cord was decompressed and fully visualized. A temporary rod that had been contoured to accommodate the deformity was applied to maintain spinal stability and avoid spinal cord injury during decompression and focal debridement (Fig. 1e). The posterior elements, including the spinous processes, laminae, facet joints, and transverse processes on the working side of the vertebral column (opposite to the side with the temporary rod) were removed to expose the lateral wall of the pedicle. Once the lesions were located in the thoracic and intercostal nerve roots, the spinal branch of the segmental artery was safely sacrificed by ligation. The ribs at the apex level were transected 3–4 cm lateral to the costotransverse joint, and the transverse process and the pedicle were excised. The nerve roots of the affected lumbar level were gently retracted to make room for the operative procedure of anterior decompression or debridement, as described by Rajasekaran et al. [10]. All infected and necrotic disc and bony tissue were resected until fresh bleeding bone was reached. Paravertebral and epidural abscesses were drained, and debridement was meticulously performed until there was no pus or infectious debris. Extreme caution was maintained during debridement near the medulla spinalis, and curettes and rongeurs were used very gently. The same process was carried out on the opposite side of the lesion if necessary. The rods were tightened, and the kyphosis was slowly and carefully corrected with the help of the compression and stretching of the internal fixation instrumentation. Two or three preprocessed TMCs that were filled with autogenous or allograft spongy bone were inserted in the bone trough for reconstructing the spine (Fig. 1f–i). Streptomycin 1.0 g and isoniazid 0.2 g were administered locally. A drain was placed and incisions were sutured. Resected specimens were collected for bacterial culture and pathological diagnosis.

### Postoperative care

The drainage tube was removed after 72 h unless there was an extensive paraspinal abscess or flow injection abscess, when it was left indwelling until the amount of drainage was less than 30 ml/24 h. Patients continued oral HREZ chemotherapy postoperatively. Pyrazinamide was discontinued at 6 months. Patients continued on 9- to 12-month regimens of HREZ chemotherapy. Ambulation with a brace was allowed 6–8 weeks after surgery. Patients carried out non-weight-bearing daily activities until there was radiographic or CT evidence of fusion. Patients then returned to normal weight-bearing activity.

### Statistical analysis

All statistical analyses were performed with SPSS version 19.0 statistical software (SPSS, Inc., Chicago, IL, USA). The paired *t* test was used to analyze data. Discrepancies in normal distribution were analyzed using Wilcoxon’s rank sum test, with a significance level of 0.05.

## Results

Laboratory testing found an average preoperative elevation in erythrocyte sedimentation rate (ESR) of 49.27 ± 12.13 mm/h (range 13.2–112.1 mm/h) (Table 1).

The duration of the single-stage posterior surgery averaged 2 h and 15 min (range 1 h and 45 min to 2 h and 30 min). Blood loss averaged 435 ml (range 250–600 ml), and the average hospitalization time was 10.4 days (range 7–15 days). TB was confirmed by bacterial culture or pathological diagnosis for all patients, and eight patients were positive for mycobacterium. A total of 59 preprocessed TMCs were implanted in 28 patients; two TMCs in 25 patients and three TMCs in three patients). All of the TMCs were thoroughly fused, with fusion time ranging from 6 to 12 months (mean 8.8 months). No implant failure or recurrence of infection was found in any patient.

The pain VAS dropped significantly from 6.31 ± 1.25 preoperatively (range 4 to 9) to 2.17 ± 1.32 postoperatively (*p* < 0.05), then to 0.57 ± 0.14 at the final follow-up (*p* < 0.05) (Table 1). The ODI scores dropped significantly from a preoperative 39.14 ± 12.38 to 7.29 ± 3.09 at 1 year postoperatively and 6.77 ± 2.53 at final follow-up (all *p*’s <0.05) The ESR returned to normal in all patients by the final follow-up (Table 1).

The sagittal profile was corrected in all cases (Figs. 1, 2, 3, and 4). The average preoperative Cobb’s angle was 44.32° ± 7.26°, which decreased significantly to 5.41° ± 0.63° postoperatively (*p* < 0.05). The average Cobb’s angle was maintained at 5.86° ± 0.57° at the final follow-up, without obvious loss of correction compared with the postoperative Cobb’s angle (*p* < 0.05) (Table 1). The ASIA grade for the nine patients who showed incomplete neurologic lesions before surgery was significantly improved after surgery; seven of these patients had returned to normal by the final follow-up (*p* < 0.05) (Table 2).

## Discussion

The conditions associated with spinal TB include the presence of a neurologic deficit, abscess formation, persistent or recurrent infection, severe pain, local kyphosis, and segmental instability. Surgical intervention offers immediate relief of severe pain and improvement of sagittal balance, particularly in thoracic and lumbar spinal TB. It also improves neurologic impairment, thus allowing early ambulation [11–13]. In the present study, intractable pain was evidently remitted by surgery, and incomplete neurologic function in nine patients improved significantly (Table 2); patients carried out their normal daily activities while wearing a brace just 7 days after surgery.

Several surgical treatment methods for spinal TB have been reported in recent years, including the anterior approach and staged or simultaneous anterior decompression combined with posterior stabilization [14–16]. As anterior debridement and interbody fusion offer the most direct approach for adequate decompression and reconstruction of the spine, some surgeons have advocated that anterior debridement and interbody fusion combined with posterior instrumentation is the ideal method for treatment of low thoracic and lumbar spinal TB [6, 17].

However, the combined anterior-posterior method has a longer operation time, greater blood loss, and longer hospitalization compared with the posterior approach alone [18, 19].

A large number of studies on the single-stage posterior approach for spinal TB treatment have reported good clinical efficacy [20–23]. Zhang et al. reported one-stage posterior debridement and instrumentation for treating young and older patients with thoracic or lumbar TB; the grafted bones were fused within 10 months in all patients, the kyphotic angle was significantly corrected after surgery, and there were no relapses during long-term follow-up [19–21]. Sahoo et al. reported 18 select cases of thoracolumbar spinal TB treated by posterior-only approach surgery [16]; kyphosis was improved from 17.7 ± 5.8° preoperatively to 9.4 ± 4.6° postoperatively, 55.5 % of cases achieved bony fusion by final follow-up, and the Cobb’s angle was corrected from 22.31° ± 4.26° preoperatively to 5.86° ± 0.57° at final follow-up [16].

We applied specially formed TMC interbody bone graft technology designed specifically for a single one-stage posterior surgery. The design of the TMCs (usually two) is made appropriate to the size of the anterior column defect space after debridement; then, the TMCs are implanted in the anterior column through the channel after debridement; this reduces the difficulty during TMC implantation and also ensures the strength of the bone graft. The TMC design not only includes the transverse section design according to the particular shape for the bone graft but also includes the coronal and sagittal section designs according to the length of the bone defect.

Autograft bone is a better substitute material for reconstructed spine than allograft bone; however, donor site complication rates as high as 10 % have been reported after autogenous bone grafting. Chronic donor site pain has been reported in up to 40 % of cases [24, 25]. More importantly, both autograft and allograft bones are associated with the complication of fracture during the operation and postoperatively [26, 27]. To the best of the authors’ knowledge, no previous clinical studies have addressed the use of TMCs in a posterior-only approach. In the current study, we conducted posterior debridement, then used posteriorly implanted TMCs that were filled with autograft or allograft bone, then combined this with instrumentation reinforcement. The average Cobb’s angle was improved from 44.32° ± 7.26° preoperatively to 5.41° ± 0.63° postoperatively without obvious loss of correction by the final follow-up (Figs. 1, 2, 3, and 4).

TMC-related complications with device fracture are relatively uncommon [27], with only few instances reported previously in the literature [26, 27]. This was verified in our study results.

TMCs offer several advantages, including immediate anterior stabilization, approximation of the intervertebral disc height, and obviation of bone graft harvesting outside the surgical site [14, 17]. A previous meta-analysis found that posterior instrumentation increased the average fusion rates with TMC interbody devices to 95 % of patients [28]; this was higher than the fusion rate reported by Sahoo et al. [16]. Furthermore, bony fusion rates with TMCs are significantly higher than autograft and allograft bone fusion rates (95–100 vs. 89.7 %) [28, 29]. Although subsidence was the most frequent complication for TMC use, Epari et al. theorized that fusion may be assisted in the TMCs by their inevitable subsidence [30]. When the intervertebral height decreases slightly, the load on the bone graft within the cage increases, which contributes to bone healing. In our study, all of the TMCs were thoroughly fused, with an average fusion time of 8.8 months.

Implantation of instrumentation in the setting of a spinal infection has been controversial. Lee et al. affirmed that TMCs have gained acceptance in reconstructive surgery performed in the setting of concomitant infection [31]. The adherence and biofilm formation of *M. tuberculosis* were evaluated on various spinal implant surfaces; *M. tuberculosis* was rarely adhered to metal surfaces and showed scanty biofilm formation, which provides the basis for successful implantation and instrumentation in TB lesions [32].

Previous studies have reported that laminectomy, partial resection of the facet joints, and the moderate stretch of the nerve root provide adequate surgical space and visualization for the removal of the sequestra, collapsed vertebrae, intervertebral discs, and para-spinal abscesses [14, 19, 20, 22]. Meanwhile, residual *M. tuberculosis* can be effectively eliminated by saline irrigation at the lesion site, local administration of anti-TB drugs intra-operatively, and postural drainage postoperatively. The posterior-only approach has advantages in surgery time, volume of bleeding, and hospitalization, and posterior fixation can be achieved without changing the patient’s position.

However, the posterior-only approach poses great challenges for surgeons. First, the spinal cord must be meticulously protected during laminectomy, partial resection of the facet joints, and debridement, especially in patients with thoracic TB or thoracolumbar TB. Zhang et al. reported two patients’ neurologic deficits were aggravated during excessive stretching of the S1 nerve [21]. Second, the TMCs should be preprocessed before being inserted in the bone trough for reconstructing the spine (Fig. 1f, g). The normally circular TMCs could not fit through the relatively limited space to the anterior column; however, smaller TMCs cannot provide enough strength and contact surface for bone fusion. Hence, we transformed the circular TMCs to special-shaped TMCs, which not only reduced the space needed for TMC implantation but also efficaciously decreased the influence on the spinal cord and surrounding structures. In the present study, no patients experienced worsening of neurologic dysfunction. Lastly, radiologic evaluation should determine whether the pathological vertebrae are implanted with pedicle screws (Figs. 1 and 2). Reserving more spinal motion can effectively prevent the degeneration of adjacent vertebrae and prevent back pain.

Some potential shortcomings of our study should be considered. The number of cases reported was few. To reaffirm the utility of this approach, it needs to be conducted on a larger number of cases. It would also be useful to compare the outcome with that obtained in simultaneous cases using the combined anterior-posterior approach.

## Conclusion

Debridement, interbody fusion with TMCs, and combined posterior instrumentation can be an effective and safe method for treating thoracic and lumbar spinal TB via posterior-only approach. This method can thoroughly debride the focus of infection, improve neurological function, and reconstruct the spinal stability. The presence of the TMCs anteriorly at the site of the infection focus had no negative influence on the course of infection healing; it also stabilized the affected segment maintaining sufficient sagittal profile. Meticulous surgery combined with a standard regimen of chemotherapy is the key point to the early eradication of spinal TB and minimization of surgical complications.
